# Editorial: Effective and attractive communication signals in social, cultural, and business contexts

**DOI:** 10.3389/fpsyg.2023.1205329

**Published:** 2023-05-16

**Authors:** Oliver Niebuhr, Francesca D'Errico, Anna Esposito, Ellen Schmid, Alexander Brem

**Affiliations:** ^1^Centre for Industrial Electronics, University of Southern Denmark, Sonderborg, Denmark; ^2^Department of Educational Sciences, Psychology and Communication, University of Bari ‘Aldo Moro', Bari, Italy; ^3^International Institute for Advanced Scientific Studies (IIASS), Department of Psychology, Università della Campania “Luigi Vanvitelli”, Naples, Italy; ^4^Department of Business Administration, Universität der Bundeswehr München, Neubiberg, Germany; ^5^University of Stuttgart, Institute of Entrepreneurship and Innovation Science (ENI), Stuttgart, Germany; ^6^Department of Technology and Innovation, University of Southern Denmark, Sonderborg, Denmark

**Keywords:** charisma, speech, gestures, leadership, branding (marketing), E-commerce, emotion, social media

According to current social media statistics, the number of YouTube users, including influencers, has doubled worldwide since 2017 (Gandola, 2022). During the period of our Research Topic alone, about 300 million people joined YouTube to consume and produce content. The number of podcasts and their listeners is growing constantly by around 20% for years now as well, prompting Richter (2022) to ask whether podcasts could become a new mass medium. A recent survey among executives of leading companies in 10 Western countries found 10 years ago already that almost 80% of them support having a business-related social media presence (Gesenhues, 2013). In fact, more than 70% of CEOs, like those from the German DAX-40 companies, regularly post on social media, especially LinkedIn, and use it more and more strategically, in this way becoming “corporate influencers” (Warschun and Zehnder, 2020). Finally, voice assistants and related talking machines are on their way to conquer our everyday lives. Since the start of our Research Topic, their number has almost doubled to an estimated 8.4 billion units worldwide (Laricchia, 2022). It is impressive statistics like these that motivated us to create the Frontiers Research Topic (RT) on effective and attractive communication signals.

The common idea behind our initiative was that communication signals are not only (and perhaps not even primarily) about exchanging propositions. We talk and text and listen and read not least for social reasons. Communication is essentially a social instrument; we seek attention, we define and change social hierarchies, we create and develop social bonds, and in all these contexts we share feelings, emotions, attitudes, tears, and laughter, even with voice assistants (D'Errico and Poggi, 2016; Poggi and D'Errico, 2022). Without the essentially social nature of communication, there might be no “small talk”, storytelling would not be a successful rhetorical strategy, AI's would not be able to predict the success of romantic relationships or business meetings from linguistic and phonetic features, attempts to change a language (like in the debate on gender-neutral language) would take place without sparking emotionally charged debates, and commercials, poetry, and news would probably all look and sound identical.

The decisive point of our RT in this context was that the individual scientific disciplines dealing with effective and attractive communication signals have so far researched and published largely independently of one another. Our RT therefore not only set itself the goal to advance the understanding of these signals and their effects, but also to make such original findings visible across disciplines and accessible in the form of open-access papers, in order to stimulate interactions and collaborations across disciplines. I think we have done remarkably well in achieving both of these goals!

I would like to thank our 111 authors for working so hard on their papers, and my active team of co-editors from the fields of Social Psychology (Francesca D'Errico), Behavioral Cognitive Systems (Anna Esposito), Entrepreneurship (Alexander M. Brem) and Leadership (Ellen A. Schmid) for guiding our authors so quickly and smoothly through their revision processes. Fifteen accepted papers were our original goal when the journey began in 2021. We ended the journey with 35 accepted papers—out of 55 submissions. The rejection rate of almost 40% speaks for the conscientious work of the many reviewers, to whom we are also very grateful, and it reflects the high quality and innovativeness of the remaining contributions—most of which have already achieved an above-average number of views by Frontier's standards.

In terms of content, the effective and attractive communication signals investigated in this RT range from the language of social commerce and brand passion (Bai et al.) to strategically applicable sound-meaning associations of Japanese vowels (Ando et al.) to women's intention behind wearing high heels (Masaryk et al.).

Emotions in particular, run like a red thread through the chronology and disciplines of the RT. More than half of all contributions to the RT are directly or indirectly related to the production or perception of human emotions. Let us follow this thread to illustrate how diversely and substantially the RT has advanced our understanding of effective and attractive communication signals. Beforehand, however, I would like to ask all authors for their understanding that, given the word limit of this editorial, we cannot portray all papers equally. It does not mean that we value the papers less that analyzed connections between, for example, intonation and eyebrow movements in YouTubers' realization of word stress (Berger and Zellers), between cyber-personality and liking expression (Li and Wang), between voice acoustics and motivational speech (Voße et al.), or between social media usage and consumers' purchase intention (Hu and Zhu). The conclusions that we draw from the emotion studies portrayed below actually apply in similar ways to all these other papers as well.

Liang et al. show, for example, that gaze direction significantly influences the perception of emotions. As stimuli, they used photographs of people either looking directly into the participants' eyes or avoiding eye contact with the participants. People with direct eye contact were more likely to be attributed positive emotions by participants, while avoiding eye contact was associated with negative emotions of fear or anger—notably, this was true for both smiling and neutral faces in the photos. These results are not fully consistent with previous studies. Liang et al. attribute that to the Asian culture where the experiment took place (as compared to previous experiments conducted in Western contexts). Mauchand and Pell did not examine images, but sounds, more precisely, the tone-of-voice of speakers who aim to trigger empathy in interlocutors through third-party complaints. In addition to the difference between a neutral vs. an emotional (complaining) tone of voice, the verbal content of the stimuli and the social status of the speakers were varied as well. Mauchand and Pell found only small effect sizes for influences of social status and verbal content on the interlocutors' empathy responses. That is, “what characterizes a complaint is how it is said [...], more than what it is about or who produces it”. The two researchers thus draw conclusions similar to those drawn in connection with charismatic speech (cf. Caspi et al., 2019) and even hate speech (Paciello et al., 2021; cf. Niebuhr and Neitsch, 2022).

Voice patterns, i.e. prosodies, also played a role in several other RT contributions. Pearsell and Pape analyzed the relationships between prosody and the Big-5 personality traits, specifically those traits associated with perceived speaker charisma. Unlike earlier studies, they focused on the 4th dimension of prosody, viz. timbre (see Campbell and Mokhtari, 2003). In agreement with previous studies, Pearell and Pape found that a smiling voice is positive for perceived charisma, while a creaky voice significantly undermines the speaker's perceived charisma (see also Pointer et al., 2022; Tschinse et al., 2022). Both effects were more pronounced for female than male voices, at least in combination with short single-statement audio stimuli; for longer audio stimuli this might no longer apply, see Tschinse et al. (2022).

The contribution by Trouvain and Weiss to the RT elaborates on smiling, which is becoming a hot topic in the speech sciences. Trouvain and Weiss discuss, with special emphasis on synthetic voices, how (auditory) smiles can improve audio books, social robots, and dialogue systems in the future, and what acoustic requirements have to be met to that end. Trouvain and Weiss concluded their paper with a call-to-action: before we can properly implement smiling into synthetic speech “basic research is needed with respect to (i) when exactly, (ii) to which degree, and (iii) for which purpose humans smile in spoken interaction”. In the paper by Niebuhr and Siegert, voice and technology are not viewed from a constructive but from a destructive perspective. With reference to previous perception results, the two researchers found that speakers who make cell-phone or video calls at high speech-compression rates can be subject to “digital flat affect”. That is, listeners are no longer able to recognize the emotions that those speakers intended to convey. This even applies to strong basic emotions such as happiness, anger, fear, or boredom. In their RT contribution, Niebuhr and Siegert identified the acoustic distortions underlying this “digital flat affect”. Results showed that the acoustic distortions of intonation were less responsible for the “digital flat effect” than those of timbre, which suggests that emotions are acoustically encoded in timbre patterns to an extent that has perhaps been underestimated so far, similar to what Pearsell and Pape suspect as well (see above).

Four other papers look at the voice-emotion link from a more performance-oriented angle. Based on the assumption that TED talkers are “good speakers”, Skarnitzl and Hledíková compared the vocal performance indicators of Czech and English TED talks, 10 per language. In an acoustic analysis, they found that English TED talkers had more melodic variability and paused more often than Czech ones. Skarnitzl and Hledíková discuss these findings with reference to a culture and language-specific perception of perceived speaker charisma (see Biadsy et al., 2008). Barbosa examined how the acoustics of recited poetry correlates with the perception of pleasantness and wellbeing in listeners. To that end, he made Brazilian and European Portuguese speakers read the same poem. An extensive acoustic analysis showed that it was primarily pausing (pause duration, pausing rate) and timbre (spectral emphasis and LTAS slope) that correlated with the listener ratings. The voice pitch level also played a role. Specifically, at least for poetry reading, a feeling of pleasantness and wellbeing was best conveyed by very low (or very high) and overall softer voices. Speaking slowly with long pauses also contributed to triggering pleasantness and wellbeing. By contrast, the study of Valls-Ratés et al. was about the exact opposite type of feelings: fear and stress or, more precisely, people's very widespread public-speaking anxiety (PSA). Based on a large-scale study that combined acoustic analyses and ratings, the researchers showed that, on the one hand, VR-based public-speaking training was able to trigger a PSA response similar to that of a real audience; on the other hand, only a few minutes of VR-based public-speaking training per week were already sufficient to significantly reduce the PSA level of speakers and, unlike a non-VR control group, the VR speakers also considered their training beneficial for their own performance. This evaluation was consistent with the acoustic analysis of Valls-Ratés et al., according to which the VR speakers' voices became more sonorous and melodic and their speaking rates slower and calmer after training.

From reduced anxiety, it is not far along our red thread until attractiveness and fun. Studies on such communication signals are also included in our RT. For example, Rathcke and Fuchs examined laughter in a heterosexual speed-dating setting. To that end, they arranged and recorded 12 dates between single men and women, who, in addition, also took part in same-sex conversations as a baseline condition. After each session, participants rated the interlocutor's romantic attractiveness. Rathcke and Fuchs concluded from their data that romantic attraction is indeed indicated to a certain degree by laughter and can thus be predicted by laughter. Specifically, “(a) laughs are particularly frequent in the first minute of the conversation, (b) daters who are mutually attracted show a significantly larger degree of temporal overlap in laughs, (c) specific laughter types (classified as a nasal ‘laugh-snort') prevail in high-attraction dates”. Lee and Ng also analyzed romantic attractiveness, albeit for Cantonese rather than German—and moreover, not in terms of laughter, but in terms of acoustically projected body size. Female participants rated photos of male faces for visual attractiveness and then produced short utterance sequences related to these faces. Results showed that, a higher visual attractiveness of male faces made female participants speak with less breathy, lower-pitched voices and such that the formant (i.e. resonance) frequencies in their speech were lower and closer together. That is, they spoke with an overall voice pattern suitable for conveying a larger acoustic body size. These effects were stronger the more satisfied the female speakers were with their own height. Finally, Rodero's study reiterates the emotions associated with perceived speaker charisma and additionally, comes full circle back to the study by Liang et al. as it includes visual behavioral patterns. Rodero made speakers combine different hand/arm gesture amplitudes with different degrees of melodic variation and let 120 participants rate the resulting A/V stimuli, both explicitly via rating scales and implicitly by measuring the participants' skin-conductance response (EDA). Rodero's results were consistent with Rosenberg and Hirschberg (2009), who have already pointed out the potential risk of exaggerating non-verbal parameters in the perception of speaker charisma, and Michalsky and Niebuhr (2019), who have experimentally defined these overdose thresholds for numerous prosodic parameters. Rodero also found that, perceptually, the most effective and attractive speakers were characterized by moderate levels of gesturing and melodic variability. Speakers with strongly pronounced gestures performed best in terms of EDA biosignals, but only when their melodic variability was low or moderate. In any case, speaking with strongly pronounced gestures and with a high level of melodic variability was perceived as neither effective nor attractive.

Powerful public speaking is undoubtedly closely related to commercial activities, and our RT also features emotion-related papers on such activities. For example, supporting the findings of previous studies but for a different business sector and cultural environment, Lee et al. report the following: Transformational, i.e. essentially charismatic business leaders, can positively influence their employees' job performance, and more generally, the emotional intelligence of business leaders is positively correlated with employees' trust in them and, interestingly, also in each other. Employees' trust in each other furthermore determines the extent to which the business leaders' emotional intelligence can positively affect employees' job performance. From the passion of leaders and employees, we proceed to Bai et al. and the passion for brands and how intense, highly emotional consumer–brand relationships can be established. Bai et al. showed how self-expressiveness and susceptibility to interpersonal influence contribute to creating an intense consumer–brand relationship. Specifically, based on their data, they stress that brand “managers should consider customers' desire for self-expression and their need to connect with others” and that having a “passion branding strategy” is essential for the success of a product, at least in the investigated Asian context. A particularly emotionally charged type of communication signal is humor, especially black humor. Ning et al. analyzed the influence of brand-to-brand teasing on consumer engagement, by means of systematically varied Twitter tweets in a large-scale perception experiment. They found that low-aggressive humor can increase consumer engagement for one's own brand, especially if the humor is presented with a wink and matches with the company's own brand personality.

Let us move on from good and bad humor to good and bad news. Zhang et al. studied how credit rating agencies on the one hand, and bond investors on the other, deal with good and bad news. From a large-scale field study for which almost 250,000 news articles were collected and analyzed over 10 years (2010-2020), Zhang et al. conclude that credit rating agencies and bond investors behave fundamentally differently, at least in China. While bond investors react very sensitively to bad news, credit rating agencies largely ignore such news; and, compared to credit rating agencies, bond investors react more strongly to bad than to good news, and are generally less sensitive to news from state-owned companies. All about bad news is Wei's study on failed bookings with online travel agencies. Wei empirical research shows that the higher the level of negative emotions caused by failed bookings, and the worse the booking agency reputation, the weaker the service recovery satisfaction is for customers. In practical terms, this means that online travel agencies must act quickly after a failed booking in order to prevent negative emotions building up in customers, and they must increase their reputation in order to have a loyalty buffer with customers. Media also plays a role in the study by Li and Zhong. Based on field data, the two researchers evaluate the media's influence on risk perception, emotions and behavior of the Chinese population during the COVID-19 pandemic. Their results showed, in the form of correlation analyses, “that the degree (frequency) of people's use of different media in…COVID-19…significantly affected their negative emotions of fear, worry, and anxiety.” The data showed that negative emotions were more readily influenced by online media as compared to “interpersonal and television [influences], while positive emotions were mainly influenced by people's use of television”.

The study by Scardigno et al. was motivated by the COVID-19 pandemic as well. The aim was “to shed light on the communicative features of the charismatic leadership of Pope Francis during the pandemic emergency” by means of analyzing both his discursive rhetorical figures and “his multimodal body signals in the global TV event of the Universal Prayer with the Urbi et Orbi Blessing”. Scardigno et al. reveal and define Pope Francis' “humble charisma”. He combines open posture and inclusive wording (e.g. the use of “we”) with emotionally charged language and common rhetorical figures such as metaphors and tri-part lists on the one hand, and with expressions of weakness and humility on the other (D'Errico and Poggi, 2019). Finally, the last study portrayed here by Mastropietro et al. is also about humility; or rather, authenticity, because what the researchers showed in their cross-modal perception experiment with 175 participants was that Obama's humble charisma (determined in previous studies) was only effective and rated as authentic if his facial expression—varied in terms of photos—was emotionally consistent with the content of speech excerpts read by the participants. In the case of mismatches, Obama's facial expression was considered “simple socially desirable posturing”.

Given all these RT studies that revolve around the production and perception of emotions, what fundamental conclusions can we draw about the nature of the communication signals analyzed? Clearly, the RT underpinned that the communication signals we all send out are (beyond their mere information content), always more or less effective and attractive. Communication signals, be they written or spoken, can determine what we buy, who we like, who we trust and forgive, whether we feel or overcome fear, and who we pay attention to commercially or socially. The signals are multimodal and to some extent both similar and different across cultures and languages. They can be positive and negative, with negative signals not necessarily evoking negative or unwanted reactions. For instance, speakers can come across as charismatic even if they convey righteous anger. In addition, culture and language-specific overdose thresholds exist and must be defined and taken into account. Related to this, it is apparent that effective and attractive communication signals must be internally consistent in order to unfold their (intended) effect on recipients. All of the above implies that effective and attractive communication signals are worth being trained and that they need to be well mastered. This applies not only in social contexts, but perhaps even more in economic and political contexts.

Looking ahead, given the multi-channel nature and contextual complexity of effective and attractive signals, such good mastery seems a long way off, both in theory and in practice, and particularly for voice assistants and other talking machines. As was stated by Trouvain and Weiss, we first have to better understand how these signals are used among us humans before we can—context-sensitively—identify patterns and transfer them to machines (Castellano et al., 2021). There is still a lot to learn and to research, especially regarding the links between communication channels (text, speech, ”body language“) and the timbre of voice acoustics (which includes laughter and smiles). The same applies to the digital technology that we create and use to transmit our effective and attractive communication signals and to support our training of these signals, for example by means of VR.

My co-editors Francesca D'Errico, Anna Esposito, Ellen A. Schmid, Alexander M. Brem, and I hope that this RT can make a lasting contribution to connecting the relevant scientific disciplines with regard to effective and attractive communication signals, both in terms of their exploration as well as in terms of their quantification, evaluation, and training.

The English satirist Charlton Brooker once said that, “in the age of social media, everyone's a newspaper columnist”. In a similar context, the US American author and speaker pointed out that “we aren't in an information age, we are in an entertainment age”. It is up to us researchers, designers, and developers of effective and attractive communication signals to live up to the implicit responsibility expressed in these quotes. I think we are—together—on an effective and attractive way to achieve this goal.

## Author contributions

ON authored the editorial on behalf of all co-editors. ON, FD'E, AE, ES, and AB contributed equally to the conception, design, and organization of the Research Topic. ON, FD'E, AE, and ES additionally edited and/or reviewed manuscripts for the Research Topic. ON and FD'E are furthermore co-authors of manuscripts published in this Research Topic. All authors contributed to the article and approved the submitted version.
